# Self-Adaptive Framework Based on MAPE Loop for Internet of Things †

**DOI:** 10.3390/s19132996

**Published:** 2019-07-07

**Authors:** Euijong Lee, Young-Duk Seo, Young-Gab Kim

**Affiliations:** 1 Department of Computer and Information Security, Sejong University, Seoul 05006, Korea; 2 Department of Data Science, Sejong University, Seoul 05006, Korea

**Keywords:** self-adaptive software, game theory, finite-state machine (FSM), Nash equilibrium, Internet of Things (IoT)

## Abstract

The Internet of Things (IoT) connects a wide range of objects and the types of environments in which IoT can be deployed dynamically change. Therefore, these environments can be modified dynamically at runtime considering the emergence of other requirements. Self-adaptive software alters its behavior to satisfy the requirements in a dynamic environment. In this context, the concept of self-adaptive software is suitable for some dynamic IoT environments (e.g., smart greenhouses, smart homes, and reality applications). In this study, we propose a self-adaptive framework for decision-making in an IoT environment at runtime. The framework comprises a finite-state machine model design and a game theoretic decision-making method for extracting efficient strategies. The framework was implemented as a prototype and experiments were conducted to evaluate its runtime performance. The results demonstrate that the proposed framework can be applied to IoT environments at runtime. In addition, a smart greenhouse-based use case is included to illustrate the usability of the proposed framework.

## 1. Introduction

The technology of Internet of Things (IoT) is increasingly being developed owing to its growing popularity. IoT connects several objects and can dynamically create various environments [1,2]. Certain IoT environments include several requirements that must be dynamically satisfied at runtime (e.g., smart greenhouses, smart homes, emergency handling systems, healthcare applications, sports applications, and reality applications). Therefore, to support a dynamic IoT environment, an IoT framework must execute appropriate decisions [3,4]. In this context, self-adaptive software can be applied to dynamic IoT environments because it alters its behavior or structure in dynamic environments at runtime [5,6]. In addition, there have been several studies on the application of self-adaptive software in various IoT environments such as inventory management systems [7], smart rooms [8], healthcare [9,10], middleware [11], emergency handling systems [12], smart city [13,14], and IoT networks [15,16,17]. However, each study has its own aspects, distinct characteristics, and target domains. Game theory can also be employed for decision-making in IoT environments with various requirements. Game theory is a mathematical model of decision-making between different stakeholders [18,19]. It is employed in economics, biology, and computer science [19,20,21,22,23,24]. Game theory helps to achieve an optimized decision and, therefore, it is used in various applications involving decision-making in the field of computer science [19,21,23,25,26,27]. Accordingly, game theoretical methods can be used in IoT environments to determine the optimal decision for different requirements.

In this study, we focus on the centralized IoT environment, which is an IoT distribution pattern. The centralized IoT collects data from sensors and the collected data are processed in the central process [28]. Especially, we focus on the features of system design to model various IoT environments, including verification and decision-making at runtime. In addition, we propose a self-adaptive framework that can be operated in low-computing devices. Therefore, the proposed approach can be applied to smart homes and smart greenhouses. The proposed framework is based on MAPE loop, which is a prominent control loop to organize self-adaptive software using four components: Monitoring, Analysis, Plan, and Execute [5,29]. In addition, to address the limitations associated with system design, we propose a self-adaptive software framework with finite-state machine design and a game theoretical decision-making method. The finite-state machine design is based on the designs presented in previous works (i.e., the RINGA framework [1,30,31]), while the strategy extraction method is based on game theory (i.e., the Nash equilibrium). We performed empirical experiments in various types of hardware environments and obtained reasonable results indicating that the proposed approach can be applied at runtime. In addition, an IoT-based greenhouse case study was performed to demonstrate the usability of the proposed approach.

The remainder of this paper is organized as follows. Section 2 presents a background on self-adaptive software with IoT and game theory, and includes related work describing self-adaptive software as a finite-state machine. Section 3 introduces the proposed framework. Section 4 presents the empirical evaluations. Section 5 presents a simple IoT-based greenhouse scenario. Section 6 discusses the limitations of the proposed approach and future work to overcome the limitations. Section 7 details the conclusions of this study.

## 2. Background and Related Work

### 2.1. Self-Adaptive Software Framework with Internet of Things

Self-adaptive software can detect environmental conditions and alter its behavior or structure if software requirements are violated [5]. Therefore, self-adaptive software includes a monitoring process to observe the changes in its environment and structure. In addition, it can be used to analyze symptoms associated with its environment and structure based on the monitoring data. If an adaptation is required, adaptation strategies are developed and executed. Therefore, feedback loop is crucial for the implementation of self-adaptive software. A prominent feedback loop, known as the MAPE loop, is generally used in the adaptation process of self-adaptive software and autonomic computing. The loop comprises the following four processes:

- The monitoring process is responsible for collecting and correlating data from the software and its environment.
- The analyzing (detecting) process is responsible for analyzing the adaptive symptoms by monitoring the data.
- The planning (deciding) process is responsible for determining the required changes and their execution methods (i.e., it is responsible for determining adaptation strategies).
- The executing (acting) process is responsible for applying the adaptation strategy.

Several self-adaptive software studies have employed this loop [5,6,29,30,31,32,33,34,35,36,37,38], and the proposed framework follows the MAPE loop as well. Various decentralized MAPE loop patterns have been studied for self-adaptive software such as coordinated control, information sharing, master–slave, regional planning, and hierarchical control [29]. In addition, the decentralized loop patterns are combined with IoT distributed patterns such as centralized, collaborative, connected intranets, and distributed [28]. In this paper, we propose a type of self-adaptive software based on MAPE loop with a master–slave pattern.

Various IoT studies that focus on self-adaptive software have recently been conducted. Zhang et al. [7] proposed an inventory management system for a warehousing company with a self-adaptive distributed decision support model. The proposed inventory management system was simulated with inventory management scenarios using IoT technology. Lunardi et al. [8] presented a decision support IoT framework with existing rule-based decision management systems (i.e., COMPaaS [39] and COBASEN [40]); the framework was used to support the discovery and selection of IoT devices for IoT applications. This paper does not directly mention self-adaptation. However, a loop with an update mechanism was used as the rule-based reasoner. Mezghani et al., developed a set of autonomic cognitive design patterns associated with the process of designing and developing a complex IoT-based system. The proposed design patterns employed a smart monitoring system case study for the management of patient health evolution based on wearable devices. Ouechtati et al. [11] presented an access control middleware for IoT, which can adapt to the changes in its environment. The middleware includes a dynamic adaptation process of access control rules that satisfy the requirements of IoT environments. Shekhar and Aniruddha [13] proposed a dynamic data driven cloud and edge system ($D^{3}$CES) to realize adaptive resource management. $D^{3}$CES comprises a feedback-based algorithm. The authors also developed a benchmark framework for a cyber-physical system and an IoT application.

MAPE loop-based approaches also exist for IoT technologies [9,12,41]. Muccini et al. [12] surveyed IoT distribution patterns and self-adaptation, and combined them in terms of their specific characteristics. In addition, Muccini et al. proposed an IoT modeling framework for an emergency handling system based on the MAPE loop. The framework was simulated with an IoT-based forest monitoring system. Azimi et al. [9] presented a MAPE-K loop-based hierarchical computing architecture (HiCH) for IoT-based health monitoring systems. The MAPE-K loop is a MAPE loop with shared knowledge. However, the architecture of HiCH is suited for hierarchical partitioning and machine learning-based data analysis. In addition, HiCH was employed in a case study to monitor the detection of arrhythmia in patients. Ribeiro et al. [41] designed MAPE loop-based management architecture patterns for an adaptation system in IoT environments. A self-protecting architecture based on architecture patterns was developed and the results of the implementation demonstrate that less memory was consumed, as compared to previous studies [42]. Welsh et al. [43] proposed a self-adaptive system with goal-based requirement models to ensure self-explanation at runtime. Self-explanation helps diagnose, understand, and explain emergent behavior using simply-structured domain-specific language. Beal et al. [44] employed aggregate programming to simplify the engineering and coordination of services in dynamic IoT environments. Aggregate programming focuses on ensuring the simplified design, creation, and maintenance of IoT systems. In addition, an aggregate programming approach was demonstrated in the Alchemist simulation [45], which is an extensible meta simulator for pervasive computing. A service-based Internet of Service (IoS) framework with a service model design was used to support automation planning (i.e., ASTRO-CAptEvo) [46]. ASTRO-CAptEvo uses a service model based on several characteristics that are stateful, non-deterministic, and asynchronous. The framework was employed in a car logistics scenario and demonstrated its adaptability in planning. Sylla et al. [47] designed a self-adaptive framework for reliable multiple autonomic loops. The loops are based on MAPE-K and comprise three patterns (i.e., parallel, coordinated parallel, and hierarchic). In addition, each loop was designed based on the composition of automata-based controllers. Renart et al. [47,48] proposed a framework to support dynamic data driven IoT applications called Pulsar. Pulsar leverages edge resources to support location and content aware processing of data streams. Pulsar was designed using an extended associative rendezvous (AR) interaction model [49] to support workflow topologies of data streams. In addition, Pulsar comprises three different layers: infrastructure, federation, and streaming.

We summarize the previous studies and the proposed framework in Table 1. However, each of these studies includes its own aspects and distinct characteristics in various platforms and IoT environments. In this paper, we aim to support the IoT environment that includes several requirements, sensors, and actuators with central processing. Therefore, we focus on modeling, verification, and decision-making for the target IoT environment at runtime in low-computing devices. The modeling and verification are based on previous research [30,31], and the decision-making method is based on game theory. Details associated with modeling, verification, and decision-making are presented in Section 2.2 and Section 2.3.

### 2.2. Self-Adaptive Software Modeling by Finite-State Machines

In this paper, a finite-state machine model based on our previous studies [30,31,38] is used to describe and verify the self-adaptive software. Lee et al. proposed a framework with finite-state machine to describe self-adaptive software (i.e., SA-FSM) in the RINGA framework [31]. The finite-state machine is translated as an abstracted model in the form of an equation for runtime verification (i.e., A-FSM). The translation process comprises abstraction algorithms that are based on state elimination. The abstracted model is used for runtime verification within the MAPE loop. The framework exhibits reasonable experimental results and provides a guideline for modeling the self-adaptive software based on finite-state machine models. The aforementioned study is modified in the present study and its basic models are introduced in this section.

SA-FSM includes four types of states (satisfied, dissatisfied, adaptive, and normal) and two transitions (normal and adaptive). The satisfied state is a set of end states in which the software requirements are satisfied. In contrast, the dissatisfied state is a set of end states in which the software requirements are not satisfied. The adaptive state is a set of states in which an adaptive activity can be performed. The normal state comprises a set of states that do not affect software adaptation and termination. In addition, an adaptive transition is an adaptive strategy trigger. Therefore, when the software reaches the dissatisfied state, the adaptive strategy is triggered if it can be implemented. Based on the state and transition types, SA-FSM can be described as follows:

SA-FSM is a tuple (S, →, $s_{0}$, AP, L), where,

- S is a set of states
- S consists of four subsets: {$S_{normal},S_{sat},S_{dis},S_{adaptive}\}\subseteq S$
- →⊆S×S is the transition relationship, and it is classified into two types: $\{\to_{normal}$ and $\to_{adaptive}\}$
- $\to_{adaptive}\subseteq$$\left\{S_{dis}\times S_{adaptive}\right\}$
- $s_{0}$ is the initial state
- AP is a set of atomic propositions
- L: S $\to 2^{AP}$ is a labeling function ($2^{AP}$ denotes the power set of AP)

In the RINGA framework, SA-FSM is translated into equations for runtime verification (i.e., A-FSM). An algorithm based on state elimination is employed for the translation and the equations are used to verify the self-adaptive software at runtime by the MAPE loop. A-FSM comprises four types of states (i.e., start, satisfy, dissatisfy, and adaptive) and two transitions (i.e., A-FSM and trigger). The satisfy, dissatisfy, and adaptive states have the same meaning as in SA-FSM, whereas the start state is an initial state of the A-FSM model. An A-FSM transition includes a path from the initial state to the “satisfy” or “dissatisfy” states of SA-FSM. The definition of A-FSM transition is presented below using the intuitive semantics of temporal modalities. Let m = (S, →, $s_{0}$, AP, L) denote SA-FSM, and Path(δ) denote a path that satisfies the temporal modalities δ.

(1) $$\delta_{i}=⋃Path\left(\exists ⋄S_{sat}\left(i\right)\right)$$

$S_{sat}\left(i\right)$ indicates the *i*th satisfy state of SA-FSM. Notation ⋄ indicates “eventually” (i.e., now or eventually in the future), and ∃ indicates the existence of at least one path. Therefore, “$\exists ⋄S_{sat}\left(i\right)$” presents the existing path for reaching $S_{sat}\left(i\right)$, if there is a path from the initial state to the satisfy state of SA-FSM (i.e., $S_{sat}$). The function Path(δ) denotes a path that satisfies the temporal modalities δ, and “$Path(\exists ⋄S_{sat}\left(i\right))$” presents a path from the initial state to $S_{sat}$. Finally, Equation (1) indicates all reachable paths to the *i*th state of $S_{sat}\left(i\right)$.

(2) $$\omega_{j}=⋃Path\left(\exists ⋄S_{dis}\left(j\right)\right)$$

The meaning of Equation (2) is similar to Equation (1); however, the end of the paths is not denoted by $S_{sat}$ but by $S_{dis}$. Therefore, Equation (2) indicates all reachable paths to the *j*th state of $S_{dis}$.

If set $S_{sat}$ has *n* states, and set $S_{dis}$ has *m* states, using δ and ω, the A-FSM transition is given by

(3) $$\to_{A-FSM}=\left\{\left\{\delta_{1},...,\delta_{n}\right\},\left\{\omega_{1},...,\omega_{m}\right\}\right\}$$

A-FSM transition includes a pair of $\delta_{i}$ and $\omega_{j}$. Therefore, the transition denotes the set of all reachable paths from the initial state to the states of $S_{sat}$ and $S_{dis}$ using definition of A-FSM transition. A-FSM is described below.

A-FSM is a tuple (S, →, $s_{0}$, AP, L), where,

- S is a set of states
- S is classified into three types of subsets, {$S_{sat},S_{dis},S_{adaptive}\}\subseteq S$
- → is the set of transitions, and $\{\to_{A-FSM},\to_{trigger}\}\subset \to$
- $\to_{A-FSM}\subseteq$$\left\{S_{start}\times S_{dis},S_{start}\times S_{sat}\right\}$ is the transition relationship
- $\to_{trigger}\subseteq \left\{S_{dis}\times S_{adaptive}\right\}$
- $s_{0}$ is the initial state
- AP is a set of atomic propositions
- L: S $\to 2^{AP}$ is a labeling function ($2^{AP}$ denotes the power set of AP)

The RINGA framework includes an abstraction algorithm to obtain A-FSM from SA-FSM. However, the details of the algorithm are beyond the scope of this study [30,31]. This framework yields reasonable experimental results at runtime verification with different model-checking tools. Moreover, this framework considers modeling of self-adaptive software as a finite-state machine, suggesting guidelines for modeling the finite-state machine and the runtime verification method. The concept of RINGA framework and the modeling of self-adaptive software are applied in this study. However, RINGA presents a modeling method and a runtime verification method for self-adaptive software but the modeling method presents only an abstractive level. In addition, RINGA defines adaptation strategies at design time. Therefore, a decision-making method is required to generate adaptation strategies at runtime. In this paper, we focus on designing self-adaptive software in IoT environments to improve SA-FSM and runtime decision-making using Nash equilibrium.

### 2.3. Nash Equilibrium

Nash Equilibrium was introduced by John Forbes Nash Jr. [50] and is used to analyze the results of strategic interactions among diverse decision-makers. Furthermore, every finite game has a Nash equilibrium. Therefore, if there are several decision-makers and institutions, the Nash equilibrium provides forecasts [19]. In game theory, there are non-cooperative players who participate in a game with their own strategies for different actions. The selection of strategies can affect another player’s strategy. Each player strives to achieve an outcome with the largest possible payoff. Therefore, players choose their strategies so that an outcome with maximum payoff can be realized.

If the players are in Nash equilibrium, then any player can select a better unilateral strategy. In Nash equilibrium, no one can receive a better payoff by changing strategies because each strategy provides the best response. Therefore, no players change their strategies and, consequently, the strategies are solidified [18,50]. Based on these principles, the Nash equilibrium is described as follows. Let (S,f) be a game with *n* players where,

- $S=S_{1}\times S_{2}\times \cdots \times S_{n}$ is the strategy set of profile
- Player i∈{1,⋯,n}
- $f\left(x\right)=\{f_{1}\left(x\right),\cdots ,f_{n}\left(x\right)\}$ is the payoff function
- A payoff function is evaluated at x∈S
- $x_{i}$ is the strategy profile of player *i*
- $x_{-i}$ is the strategy profile of players other than *i*
- Player *i* selects strategy $x_{i}$ resulting in strategy profile $x=(x_{1}\cdots x_{n})$, and then player *i* obtains payoff $f_{i}\left(x\right)$.
- $x^{*}\in S$ is a Nash equilibrium when $\forall i,x_{i}\in S_{i}:f_{i}\left(x_{i}^{*},x_{-i}^{*}\right)\geq f_{i}\left(x_{i},x_{-i}^{*}\right)$.

In the proposed approach, the Nash equilibrium is used to extract a strategy to adapt to an IoT environment. Details of the applications of Nash equilibrium are presented in Section 3.3.

## 3. Self-Adaptive Framework with Runtime Decision-Making Method

A self-adaptive software framework is proposed to design an IoT environment using the finite-state machine and extract an adaptive strategy using the Nash equilibrium. Section 3.1 presents an overview of the proposed method. Section 3.2 presents the modeling of the finite-state machine based on SA-FSM. Section 3.3 presents a method to extract the strategies using the Nash equilibrium.

### 3.1. Overview

**Figure 1:**
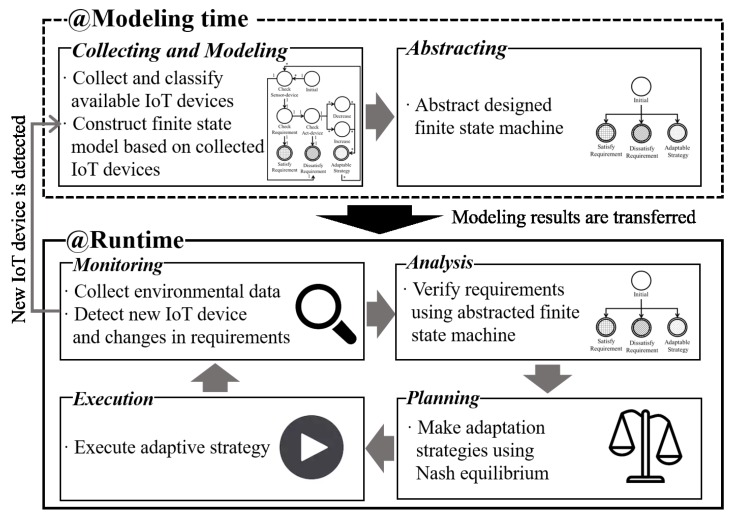
Overview of proposed framework.

In this paper, we focus on certain IoT environments consisting of several sensors, actuators, and requirements that should be dynamically satisfied at runtime. To accomplish this, a self-adaptive software framework is proposed for an IoT environment with two phases: modeling and runtime. The modeling phase is responsible for extracting the finite-state machine to describe the self-adaptive software. The runtime phase comprises a MAPE loop and is responsible for runtime adaptation. Figure 1 presents an overview of the proposed framework. As mentioned above, the modeling phase is responsible for modeling an IoT environment as a finite-state machine. The modeling phase first collects the available IoT devices and prepares a model design. The collected IoT devices are classified into two types: sensor-device and act-device. The classified devices are further categorized based on their abilities and related requirements. Details of device classification are described in Section 3.2. Following the classification, a finite-state machine is constructed using the collected IoT devices. The finite-state machine model is constructed based on the actions and relations between the collected devices. The procedures involved in modeling a finite-state machine for IoT are described in detail in Section 3.2. The final process of the modeling phase is the abstracting process. The abstracting process abstracts the designed finite-state machine using a state elimination algorithm. Details of the abstraction algorithms are described in previous studies [30,31]. It should be noted that the abstracting process is executed only once if there is no change in the design of the finite-state machine. Finally, the abstracted finite-state machine is transferred to the runtime phase. The designed and abstracted models are used to evaluate the environmental conditions of the software in each cycle of the MAPE loop.

The runtime phase is responsible for runtime adaptation. As mentioned previously, this phase comprises the MAPE loop. However, the MAPE loop can be organized into several patterns in the decentralized self-adaptive software and IoT [12,29]. The proposed framework consists of a verification method (i.e., analysis process) and a decision-making method (i.e., planning process). The verification method needs centralized data from multiple sensors (i.e., slaves for sensing) for runtime verification. In addition, the decision-making method also needs centralized data to generate the most optimal solution for adaptation. After the decision-making process, the optimal solution is executed through multiple actuators (i.e., multiple operable slaves) in executing process. Therefore, the proposed framework needs central processing for verification and decision-making, and multiple sensors and actuators to collect data and execute adaptation strategy. Therefore, the proposed framework can be classified as a centralized IoT pattern [28]. The master–slave MAPE loop pattern is appropriate for the adaptation process [12]. Therefore, one master is responsible for the analyzation and planning process, and multiple slaves (i.e., sensor-devices and act-devices) are responsible for the monitoring and executing process. The monitoring process is responsible for the collection of data that describe the environment and any internal changes. Environmental data are collected by multiple slaves (i.e., sensor-devices). In addition, the monitoring process searches a new device that has potentially been added in the finite-state machine. If a new device is detected, then remodeling is requested by the modeling process. If the remodeling request is accepted, the modeling phase executes the remodeling of the new device. Following the monitoring process, the analysis process is executed in the master. The analysis process is responsible to analyze the symptoms associated with the adaptation situation. In the proposed approach, analysis is performed by only evaluating the equations [30,31]. Consequently, the analysis process indicates that the self-adaptive software has reached adaptive states and detects the condition to adapt. The analyzed results are transferred to the planning process. The planning process is responsible for the formulation of adaptation strategies concerning the changes and their implementation. In this study, we propose a decision-making method with the Nash equilibrium, which can enable strategies to adapt to environmental changes. Details of strategy extraction are described in Section 3.3. The last process of the runtime phase is execution, which is responsible for activating the adaptive strategies. Therefore, if the planning process transfers an adaptive strategy, the executing process activates the adaptive strategy through multiple slaves (i.e., act-devices) for runtime adaptation. Subsequently, the monitoring process is executed and the MAPE loop continues.

### 3.2. Finite-State Machine Modeling for IoT Environments

We classified IoT devices as sensor-devices and act-devices for modeling IoT-based self-adaptive software. A diverse range of devices exist within IoT environments (e.g., light sensor, humidity sensor, light controller, speaker, and humidifiers). The devices can be classified into various categories. However, only two types of IoT devices are used for finite-state machine modeling: sensor-devices and act-devices.

- A *sensor-device* senses the changes in environment. Therefore, it must have embedded at least one readable sensor-device such as light sensor, humidity sensor, and temperature sensor. In addition, it is assumed that the sensor-device recognizes the requirement that is related to its sensed data.
- An *act-device* can change the environment. Therefore, it must have embedded at least one physical-device such as an LED, a servomotor, or a fan. In addition, it is assumed that the act-device recognizes the requirements that are associated with its operation.

In this study, a finite-state machine is used for modeling self-adaptive software in IoT environments, and the finite-state machine is based on SA-FSM [30,31]. However, SA-FSM is modified for IoT and it can be expressed as a tuple (S, →, $s_{0}$, AP, L), where,

- S is a set of states
- The states are classified into eight types $\{S_{sensor},S_{req},S_{sat},S_{dis},S_{act},S_{adapt},S_{inc},S_{dec}\}\subseteq S$
- $S_{dis},S_{sat},S_{adapt}$ are end states
- →⊆S×S is the transition relation, and it is classified into eleven types $\left\{s_{0}\times S_{sensor},S_{sensor}\times S_{dis},S_{sensor}\times S_{req},S_{req}\times S_{sat},S_{req}\times S_{act},S_{act}\times S_{dis},S_{act}\times S_{inc},S_{act}\times S_{dec},S_{inc}\times S_{adapt},S_{dec}\times S_{adapt},S_{adapt}\times S_{sensor}\right\}$
- $s_{0}$ is an initial state
- AP is a set of atomic propositions
- L: S $\to 2^{AP}$ is a labeling function ($2^{AP}$ denotes the power set of AP)

As indicated by the tuple definition, a finite-state machine comprises nine states and eleven transition types. The state set and related transitions include the following:

- *Initial state* ($s_{0}$) is an initial state.
- *Sensor-device state* ($S_{sensor}$) is a set of sensor-device related states. In this state, the sensor-devices must be related at least once. If a readable sensor-device is available, it reaches a *requirement state* (i.e., $S_{sensor}\times S_{req}$). However, it is connected to a *dissatisfied state* (i.e., $S_{sensor}\times S_{dis}$) if there is no related sensor-device. *Requirement state* ($S_{req}$) is a set of states that verify requirement satisfaction. If the checked requirement is satisfied, the requirement state reaches the *satisfied state* (i.e., $S_{req}\times S_{sat}$), else the *adaptive state* (i.e., $S_{req}\times S_{adapt}$).
- *Satisfied state* ($S_{sat}$) is a set of end states for which the software requirement is satisfied.
- *Dissatisfied state* ($S_{dis}$) is a set of end states for which the software requirement is not satisfied. If the finite-state machine has no readable device (i.e., $S_{sensor}\times S_{dis}$) or no possible adaptive action (i.e., $S_{act}\times S_{dis}$), the finite-state machine model reaches this state.
- *Act state* ($S_{act}$) is a set of states that check for an actable device. If there are no actable devices, the model reaches the *dissatisfied state* (i.e., $S_{act}\times S_{dis}$), else the increase or decrease state (i.e., $S_{act}\times S_{inc}$ and $S_{act}\times S_{dec}$, respectively).
- *Increase state* ($S_{inc}$) and *decrease state* ($S_{dec}$) are the sets of act-device related states. In these states, at least one actable device is related. If the finite-state machine reaches these states, the related act-device is operated. These states then reach the *adaptive state* (i.e., $S_{inc}\times S_{adapt}$ and $S_{dec}\times S_{adapt}$, respectively).
- *Adapt state* ($S_{adapt}$) is one of the end state sets that denotes the possible adaptive activities. Therefore, if the finite-state machine reaches this state, it means that the self-adaptive software must adapt and adaptive strategies do exist. In addition, this state reaches the related requirement sensor-device state to re-verify the requirement satisfaction (i.e., $S_{adapt}\times S_{sensor}$).

**Figure 2:**
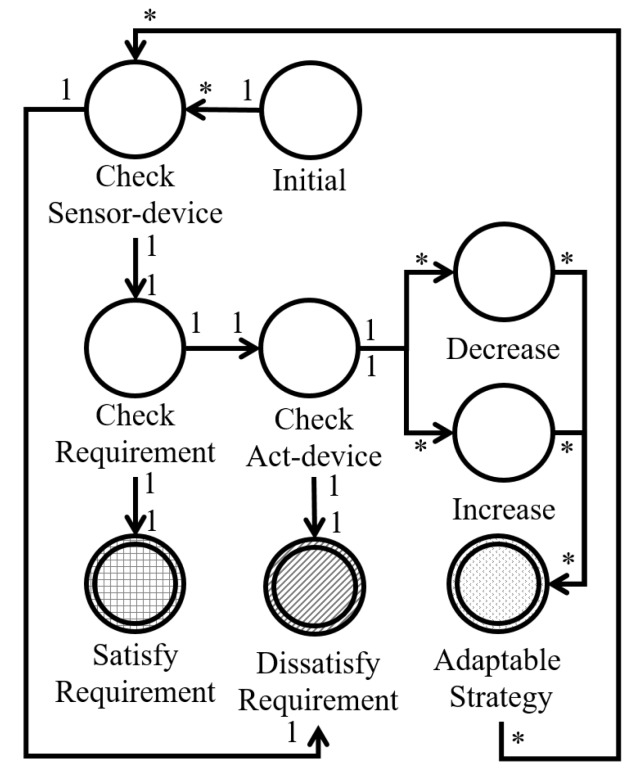
Relationship of IoT-FSM state types.

In the proposed approach, reachable paths are extracted to reach the end states (i.e., $S_{sat}$, $S_{dis}$, and $S_{adapt}$). In addition, the reachable paths are calculated at each MAPE loop to verify requirement satisfaction. Figure 2 illustrates the graphical definition of the proposed finite-state machine. As shown in Figure 2, most transitions are matched one-on-one. However, some transitions do not have one-on-one matching owing to the reason discussed below. The initial state can relate to multiple $S_{sensor}$ states, which means that the initial state can be reached by several requirements. In addition, a $S_{act}$ state can relate to multiple $S_{inc}$ and $S_{dec}$ states because multiple act-devices can exist in a single requirement. For example, if the requirement relates to illumination, several possible act-devices exist (e.g., LED, natural light). Furthermore, the states $S_{inc}$ and $S_{dec}$ can be connected with multiple $S_{adapt}$ states because the operation of an act-device can affect several requirements. For example, if an act-device can control windows, multiple requirements can be affected (e.g., light, temperature, and intensity of dust). As mentioned in Section 3.1, the finite-state machine is abstracted for runtime verification and the state elimination algorithm [30,31] is applied during modeling. There are three types of end states in the finite-state machine model (i.e., satisfied requirement, dissatisfied requirement, and adaptable state) and the abstracting process is performed for each end states. Finally, the paths from the initial state to the satisfied, dissatisfied, and adaptable states are extracted and used for runtime verification in the MAPE loop.

### 3.3. Game Theoretic Decision-Making and Evaluation

In this section, we describe the use of Nash equilibrium for strategy extraction from the finite-state machine model. Nash equilibrium is suitable for complete information game, implying that payout of each player is fully known between players. However, as described in the previous section, this paper focuses on centralized IoT environment. Thus, it is assumed that the central process already knows the operation and affection among requirements and devices in the decision-making process (i.e., the planning process in the MAPE loop). In addition, we assume that there exists only legitimate requirements and devices (i.e., there is no malicious user that interrupts decision-making); therefore, centralized decision-making can extract unbiased adaptive strategies to satisfy the most number of requirements. As described previously, an act-device can perform physical acts, affecting several requirements. In other words, a requirement can include several act-devices and the related act-devices can affect other requirements. Therefore, act-devices can be operated to satisfy requirements and the operation of act-devices can affect several requirements. In addition, if the requirements are related to overlapped act-devices, an act-device can adversely affect different requirements. In this case, one requirement may be satisfied but others may not. Therefore, the execution of strategies must effectively operate the act-devices to satisfy multiple requirements. In this context, the requirements can be considered as a player and the act-device as a strategy of the player. The Nash equilibrium for IoT is described as follows:

Let a player be a requirement and (S,f) be a game with *n* requirements, where,

- $S=S_{1}\times S_{2}\times \cdots \times S_{n}$ is the strategy set of profile
- Requirement i∈{1,⋯,n}
- $f\left(x\right)=\{f_{1}\left(x\right),\cdots ,f_{n}\left(x\right)\}$ is a payoff function
- A payoff function is evaluated at x∈S
- $x_{i}$ is an act-device profile of requirement *i*
- $x_{-i}$ is an act-device profile of players other than *i*
- Requirement *i* operates act-device $x_{i}$ resulting in strategy profile $x=(x_{1}\cdots x_{n})$ and then, requirement *i* obtains payoff $f_{i}\left(x\right)$
- $x^{*}\in S$ is a Nash equilibrium for IoT when $\forall i,x_{i}\in S_{i}:f_{i}\left(x_{i}^{*},x_{-i}^{*}\right)\geq f_{i}\left(x_{i},x_{-i}^{*}\right)$
- $x^{*}$ can be an operation candidate at runtime
- A strategy with the largest number of Nash equilibrium between the requirements is selected and implemented

Formally, if the players (requirements) reach Nash equilibrium, they cannot choose a new strategy because no one can receive a better payoff by strategy selection. However, the Nash equilibrium in the proposed model indicates candidate strategies because it denotes that there are strategies that can satisfy multiple requirements. As described above, we choose Nash equilibrium to extract candidate strategies for adaptation in runtime. However, the Nash equilibrium that can satisfy every requirement may be nonexistent if many requirements need adaptation. In this case, some strategies with the largest number of Nash equilibria can be the candidate strategies for adaptation (i.e., the last definition of Nash equilibrium for IoT) because the candidate strategies may adapt several requirements even if every requirement is not adaptable. However, there can be multiple equilibria (i.e., candidate strategies for adaptation) at the same time. Then, a method to evaluate the strategies is needed to determine the optimal strategy.

A method called *strategy score (SS)* is proposed for evaluating strategies. There are three major conditions in this method, described as follows:

- The *number of satisfied requirements (SR)* is the number of requirements that may be satisfied by the execution of a strategy. If an adaptation strategy satisfies multiple requirements, it is more efficient than an adaptive strategy that satisfies lesser requirements.
- The *number of related requirements (RR)* is the number of requirements that may be affected by the execution of a strategy. For example, if a strategy opens the windows to adjust indoor brightness, it affects humidity, dust density, or temperature. In this case, the requirements of humidity, dust density, and temperature comprise RR. It is more efficient to have fewer RRs.
- The *number of act-devices (AD)* is the number of act-devices that are executed by the adaptation strategy. It is more efficient to have a smaller value of AD.

Equation (4) presents the calculation of the SS using SR, RR, and AD.

(4) $$SS=\alpha \left\{\log\left(\frac{SR+1}{RR+1}+1\right)\right\}+\beta \left\{\log\left(\frac{1}{AD+1}+1\right)\right\}$$

The equation comprises the sum of two terms: requirement and act-device. The first term (i.e., $\log\left(\frac{SR+1}{RR+1}+1\right)$) is related to requirement. Therefore, SR and RR are used. As mentioned above, it is efficient when a strategy can satisfy several requirements (i.e., large value of SR) and affects few requirements (i.e., small value of RR). Therefore, SR is divided by RR, and it takes a logarithmic function for normalization. A value of 1 is added to prevent negative infinity output. The second term (i.e., $\log\left(\frac{1}{AD+1}+1\right)$) is related to act-devices. If a strategy can satisfy some requirements, a lower value of AD is more efficient. Therefore, a reciprocal number of AD is used and a value of 1 is added to prevent negative infinity output. The terms α and β are mediators used for adjust the power of the other two terms. This equation denotes the strategy score of a strategy within the planning process of the MAPE loop.

## 4. Experiment

A prototype of the proposed approach using JAVA 1.8.0 was implemented on different hardware environments, as listed in Table 2. As mentioned above, we focus on proposing a self-adaptive framework, with verification and decision-making, that can be operated by low computing devices, and various hardware environments are considered (i.e., server, laptop, desktop, and smart phone). The experiment aimed to evaluate the verification and decision-making performance of the proposed framework in various IoT environments.

To perform the experiment, we randomly generated IoT environments using different numbers of act-devices and requirements because, if there are more requirements and act-devices, the experimental IoT environments are more complex. In the experimental environment, the number of sensor-devices was not considered because we focused on runtime verification with abstraction process and decision-making method in this study. However, a requirement should have at least one sensor-device to detect environmental changes. Therefore, one sensor-device was assigned to each requirement of the experimental environments. In addition, each requirement included at least one act-device and the remaining act-devices were randomly assigned to the requirements. For example, when there were two requirements and five act-devices were assigned in an experimental environment, only two sensor-devices were assigned for each requirement to sense environmental changes, while two act-devices were assigned for each requirement to adapt environmental changes and the remaining three act-devices were randomly assigned for each requirement. The environment values (i.e., sensed data) were randomly varied and iterated 100 times for each experiment. In other words, we executed MAPE loops 100 times with different values. Three factors were measured: abstracting process time, analyzing process time, and planning process time. The abstracting process time illustrates the modeling time of the experimental environments; the analyzing process time denotes the verification time for a model-checking method [31]; and the planning time indicates that an optimal solution must be extracted based on the Nash equilibrium. However, we measured the time factors without initially setting the process time (i.e., JVM loading time and application loading time) because the initial setting processes were executed only once when the application started.

**Figure 3:**
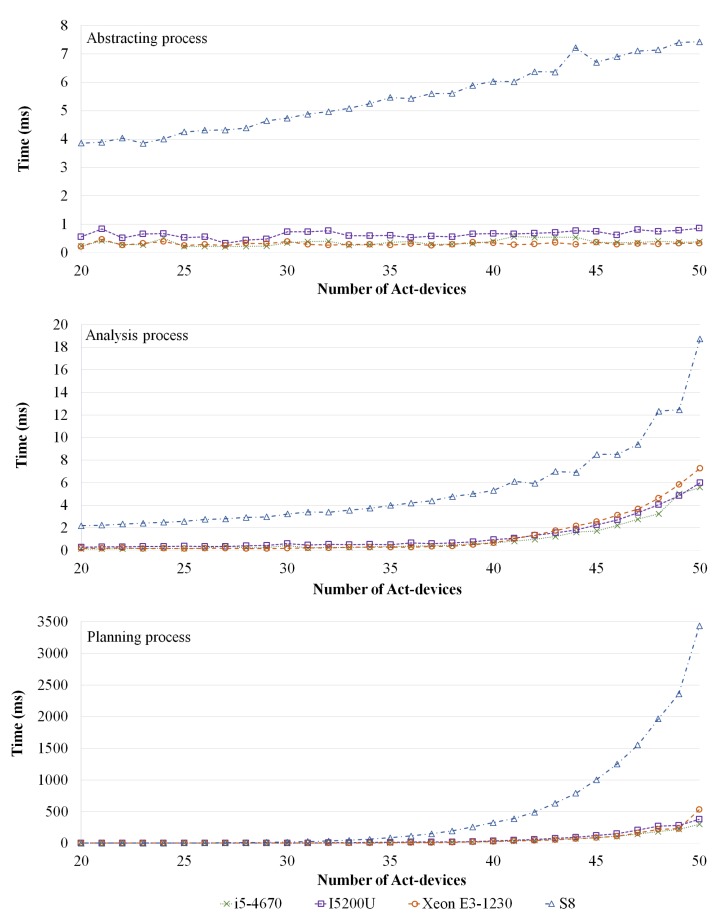
Results with fixed requirements and increasing number of act-devices.

The first experiment involved ten fixed requirements and variable act-devices (20–50). Figure 3 presents the experimental results for the increasing numbers of act-devices. As expected, the method required more time for a large number of act-devices. However, when the number of act-devices was 50, the maximum average time for abstracting the finite-state machine was less than 8 ms and the analysis time was less than 20 ms for low-computing power sources (i.e., Samsung Galaxy S8). In particular, the planning time for extracting the strategies was higher than the other methods. However, it was less than 500 ms for a high-computing environment and 3.5 s for a mobile device, even with 50 active devices. The monitoring and executing times were ignored because the prototype neither read real sensor values nor operated physical devices. Nevertheless, it was assumed that every factor was calculated within reasonable time. However, the method must be optimized for low-computing power devices (e.g., mobile device, Arduino, and Raspberry Pi).

**Figure 4:**
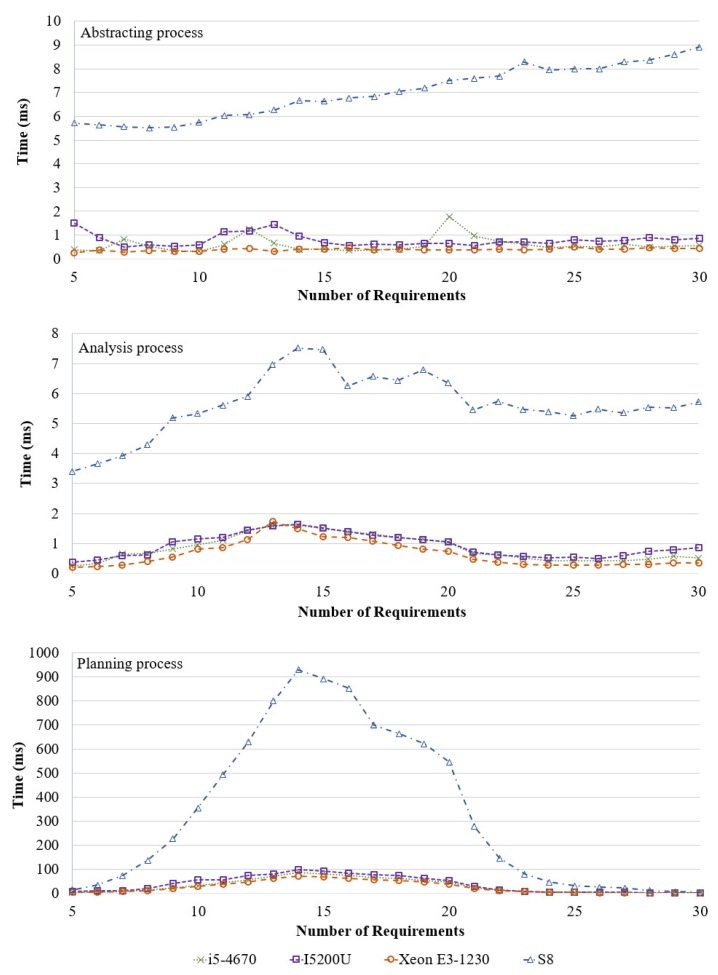
Results with fixed act-devices and increasing requirements.

The second experiment was performed using 40 act-devices and variable requirements. Figure 4 presents the experimental results. Similar to the previous experiment, the abstracting process required more time when the number of requirements was large. However, the time for analyzing and planning indicated a tendency to decrease after a rise because the interconnections between the requirements were more complicated when the requirements affected each other. To analyze the designed finite-state machine, it was abstracted to an equation (see Section 3.1). The abstracted equations indicate that all reachable paths from the initial state to the end states (i.e., satisfied state, dissatisfied state, and adaptive state), including the extraction of the reachable paths, are complex when there are many connections between the requirements via the operation of act-devices because transitions connecting the operations of act-devices (i.e., $S_{act}\times S_{inc}$ and $S_{act}\times S_{dec}$) can be connected to several requirement adaptation transitions (i.e., $S_{inc}\times S_{adapt}$ and $S_{dec}\times S_{adapt}$). In addition, if there are many connections between the act-device operations and the requirements, then there exist several reachable paths to reach the adaptation state. Therefore, the abstracted equation becomes complicated when the interconnections between the requirements from the designed finite-state machine are complicated. The results of the analysis process also demonstrate a reduced tendency after 15 requirements owing to reduced complexity because the experimental dataset has limited act-devices, which makes the requirement adaptation transitions (i.e., $S_{inc}\times S_{adapt}$ and $S_{dec}\times S_{adapt}$) less complex. In addition, to extract the Nash equilibrium, the possible actions for a requirement were compared with the possible actions for other requirements. In contrast, extracting the Nash equilibrium required less time when the interconnections between the requirements were not complicated (i.e., the result of 5 and 30 requirements in Figure 4). The reasons for this can be attributed to the results of the planning process because the verification of the relationship between the requirements (i.e., evaluating a payoff between relationships) and the possible act-device operations is required to extract the strategies. The complexity of the relationship increased when there was a significant interference between the relationship via possible act-device operations. However, the results of the planning process also show similar tendency after 15 requirements owing to a limited number of act-devices. The results of the second experiment comprehensively demonstrate that the analysis and planning times are affected by the complexity of the relationship between the requirements. Nevertheless, the second experiment demonstrated that the proposed approach is reasonable even when the requirement interconnections are complicated.

## 5. Case Study: IoT Based Smart Greenhouse

In this section, a case study is described to understand the proposed approach: IoT-based smart green house. A small-scale smart greenhouse environment was designed with three requirements, three act-devices, and six sensor-devices. It was modeled based on the proposed modeling method. In addition, three scenarios are described to illustrate the use of game theory decision-making method.

### 5.1. Overview of Case Study

**Figure 5:**
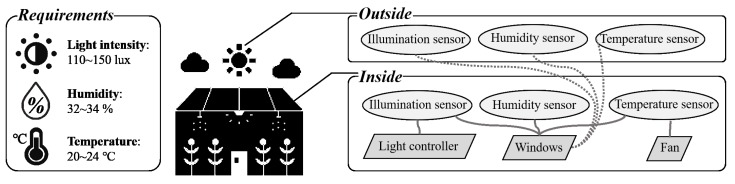
Overview of an IoT-based smart greenhouse.

The case study environment consisted of three requirements (i.e., light intensity, humidity, and temperature), three act-devices (i.e., light controller, fan, and windows) and six sensor-devices (i.e., illumination, humidity, and temperature sensor for inside and outside). Figure 5 presents an overview of the proposed case study. As shown in Figure 5, the intensity of light, humidity and temperature were considered as the requirements to provide an optimal growth environment to the crops. If the light intensity inside the greenhouse is between 110 and 150 lux, the light requirement is satisfied. Similarly, if the humidity is between 32% and 34%, the humidity requirement is satisfied. In addition, if the temperature is between 20 and 24 °C, the temperature requirement is satisfied. The status of the requirements is checked by the illumination, humidity, and temperature sensors in the greenhouse. However, there are three act-devices: light controller, fan, and window. The light controller can directly control the light intensity in the greenhouse and the fan can control the temperature by the on (falling temperature) and off (maintain temperature) states, while the window can control all requirements via the external situation to the greenhouse. For example, if the humidity requirement is to increase the humidity in the greenhouse and the humidity outside is higher than inside, the requirement can be adjusted by opening the window. Therefore, the lines that connect the sensors and an act-device (e.g., light controller and window) indicate a correlation between the connected devices. In addition, the dotted lines indicate that an act-device may control the related requirements via connected external environments (e.g., connected with dotted line).

In the case study, we assumed that the light controller was designed with an operation range between 0 and 240. The minimum value 0 implied that the device was turned off, whereas the maximum value 240 implied that the device operated at full capacity. The operation range of the lamp was 20. Thus, the lamp included 12 levels (i.e., 0, 20, 40, 60, ⋯, 240) and the operation of the light controller affected the greenhouse immediately. The fan was operated at two levels (i.e., on and off). In addition, the windows included two operations (i.e., open and close), which may affect the greenhouse based on the external environmental status. Three scenarios were created to measure the adaptability based on a game theory strategy extraction method (see Section 5.3). The scenarios included different numbers of satisfied requirements: the first scenario only included one dissatisfied requirement, the second scenario included two dissatisfied requirements, and the third scenario included all dissatisfied requirements.

- Scenario #1: The light requirement is not satisfied (80 lux), but the humidity (33%) and temperature (22 °C) requirements are satisfied. The windows are closed, and the level of the light controller is 4 (80 lux). In addition, the fan is turned off.
- Scenario #2: The light (80 lux) and humidity (31%) requirements are not satisfied, but the temperature (22 °C) requirement is satisfied. Both dissatisfied requirements must be increased for adaptation. The windows are closed, and the level of the light controller is 4 (80 lux). In addition, the fan is turned off.
- Scenario #3: All requirements are not satisfied: light (80 lux), humidity (30%), and temperature (26 °C). The light and humidity requirements must be increased for adaptation, but the temperature requirement must be decreased for adaptation. The windows are closed, and the level of the light controller is 4 (80 lux). In addition, the fan is turned off.

In addition, we designed varying external environments to demonstrate adaptability across various environments: one comprised higher environmental values (i.e., humidity, temperature, and light intensity) compared to values for requirements satisfaction (i.e., 24 °C, 34% and 150 lux), and the other comprised lower environmental values compared to values for requirements satisfaction (i.e., 20 °C, 32% and 110 lux). Therefore, each scenario was associated with each external environment, and several cases were simulated in the case study. The environments are described below.

- External environment #1: External light is brighter than the light inside (180 lux), and the external humidity and temperature are higher than that of the internal environment (36% and 30 °C).
- External environment #2: External light is darker than the light inside (20 lux) and the external humidity and temperature are lower than that of the internal environment (25% and 15 °C).

### 5.2. Modeling of Finite-State Machine

**Figure 6:**
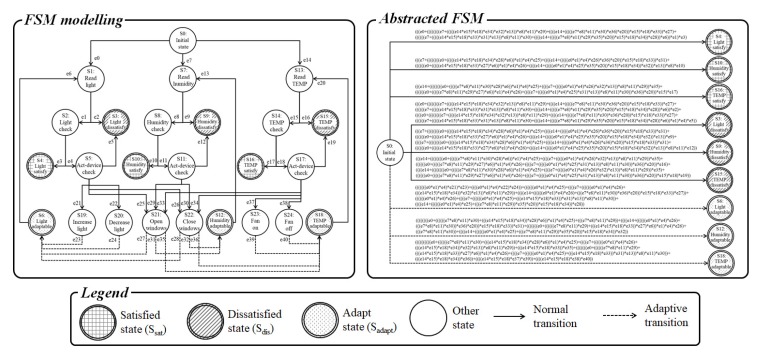
Modeling results of case study.

Based on the smart greenhouse, the finite state machine model and abstracted model was extracted, as shown in Figure 6. The finite state machine model began at the initial state (i.e., S0). There were three requirements in the case study and the status of the requirements was obtained by the sensors (i.e., S1, S7, and S13). If there was no connected readable device (i.e., e2, e9 and e16), the state of the model was dissatisfied (i.e., S3, S9 and S15) and terminated. Otherwise, the status was checked (i.e., S2, S8 and S15) and, if the requirements were satisfied, the state was terminated with after the requirement was satisfied (i.e., S4, S10 and S16). The act-devices were checked when the requirements were dissatisfied (i.e., S4, S10 and S16) and, if there were no operable act-device (i.e., e5, e12 and e19), the state ended with dissatisfaction (i.e., S3, S9 and S15). However, the state associated with checking the act-device was connected based on the operations of the act-devices (i.e., S19 to S24) and each operation state was connected with adaptable states (i.e., S6, S12 and S18) based on adaptive transition (i.e., e23, e24, e27, e28, e31, e32, e35, e36, e39 and e40). The adaptable states were connected by checking the sensor states (i.e., e6, e13 and e20) and re-checking was performed to confirm that the requirements were satisfied. The designed finite state machine model was abstracted and the equations (i.e., abstracted transitions) were extracted for runtime verification. As mentioned in the previous section, the abstracted model was used to verify the satisfaction of a requirement in the analysis process in MAPE loop at runtime. If the abstracted model reached the satisfied state (i.e., S4, S10 and S16), the requirements were satisfied. In contrast, if the abstracted model reached the dissatisfied state (i.e., S3, S9 and S15), the requirement was dissatisfied. In addition, reaching the adaptable states (i.e., S6, S12 and S18) indicated that the requirement was dissatisfied but adaptable strategies may exist. Therefore, an optimal solution was needed for adaptation when the model reached an adaptation state. The solution was extracted by the proposed game theoretic decision-making method. All transitions of the finite state machine are expressed as 0 or 1, and the abstracted transition results present the possible ways of reaching each end state [31].

### 5.3. Game Theoretic Decision Making

**Figure 7:**
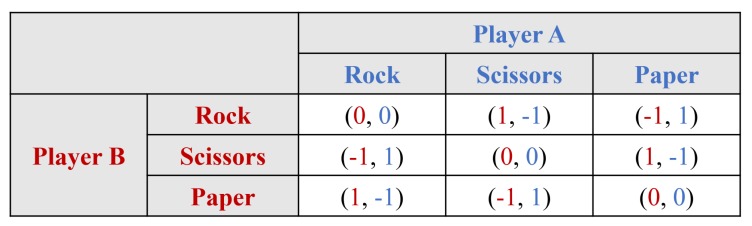
Example of a payoff matrix.

A payoff matrix is a visual representation for making strategic decisions involving two players or groups in game theory. The matrix is described with related players, strategies of the players, and the payoff results. The players are positioned in the top rows and left column, which list each player’s strategies. The payoffs of the row player are always listed as the first value in each cell. Thus, the payoffs of the column player are always listed as the second value in each cell. Figure 7 presents an example of a payoff matrix and the example describes the game “Rock, Scissors, Paper”. The results of the game are win, lose, and tie, and the payoffs are 1, −1, and 0, respectively.

**Figure 8:**
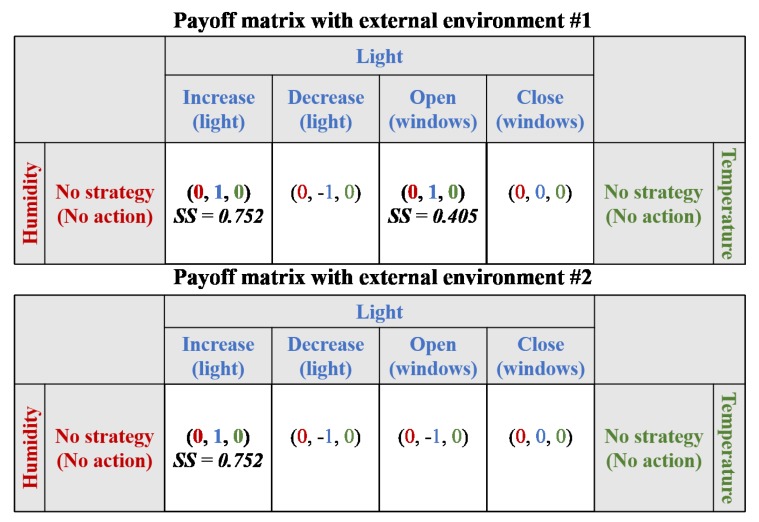
Payoff matrix for Scenario #1 (α = 0.5 and β = 0.5).

In the case study, we assumed that the results of the payoff matrix were “positive for requirement satisfaction”, “negative for requirement satisfaction”, and “no effect”, and the values were “1”, “−1”, and “0”, respectively. In the first scenario, a payoff matrix was presented with a strategy score of the external environments, as shown in Figure 8. In this scenario, only the light requirement was not satisfied, and, therefore, strategies associated with the light requirement were considered in the payoff matrix. In the first external environment, there were two strategies that could be used for adaptation within the Nash equilibrium: “increase light using light controller” and “open windows”. Both strategies involved the operation (i.e., AD in strategy score) of one device, but the former had fewer related requirements (i.e., RR in strategy score). In other words, there was a possibility that the latter may affect the humidity and temperature requirements. Therefore, the former strategy exhibited a higher score than the latter. Thus, “increase light using light controller” was selected as the optimal solution. However, in the second external environment, the scenario included only one strategy: “increase light using light controller”. Therefore, the strategy was chosen as the optimal solution.

**Figure 9:**
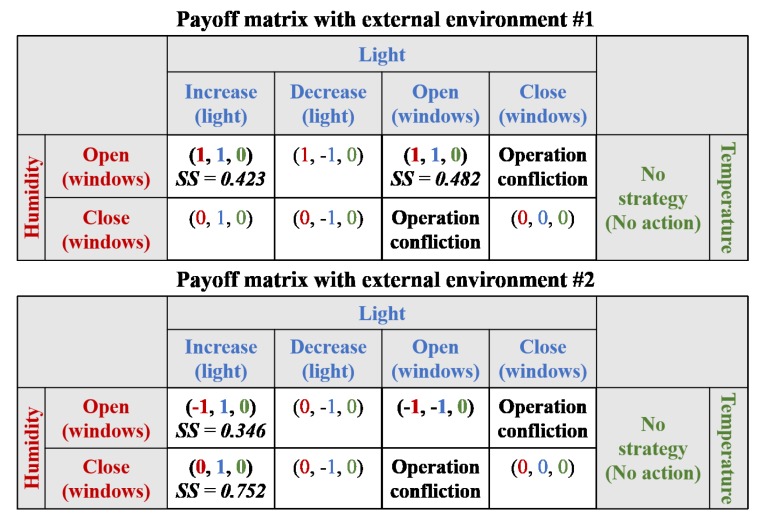
Payoff matrix for Scenario #2 (α = 0.5 and β = 0.5).

In the second scenario, two requirements were not satisfied (i.e., light and humidity requirements), and a strategy was needed to adapt to the requirements. A payoff matrix is presented in Figure 9. There were two conflicting operations (i.e., open windows and close windows), which were discarded from the payoff matrix. However, in the first external environment, there were two strategies within the Nash equilibrium: “increase light controller and open windows” and “only open windows” (i.e., both requirements activate opening the windows). Both strategies satisfied the requirements and, therefore, the strategy score was calculated to determine the optimal solution. Considering the results of the strategy score, the latter (i.e., only opening the windows) was selected as the optimal solution because the optimal solution could satisfy both requirements by activating one act-device (i.e., windows). The other strategy could also satisfy both requirements, but the strategy required the operation of two act-devices for adaptation. In the second external environment, there was no solution that satisfied both requirements, and thus there was no Nash equilibrium between both requirements. Therefore, strategies with the largest number of Nash equilibrium were ideal candidates for adaptation: “increase light controller and open windows” and “increase light controller and keep windows closed”. Both candidate strategies satisfied only the light requirement, but the latter activated only one device (i.e., the light controller was activated, and the windows was already closed when the scenario began). In addition, the latter included fewer related requirements (i.e., RR in strategy score) than the former. Therefore, the strategy “increase light controller and keep windows closed” was chosen as the optimal solution.

**Figure 10:**
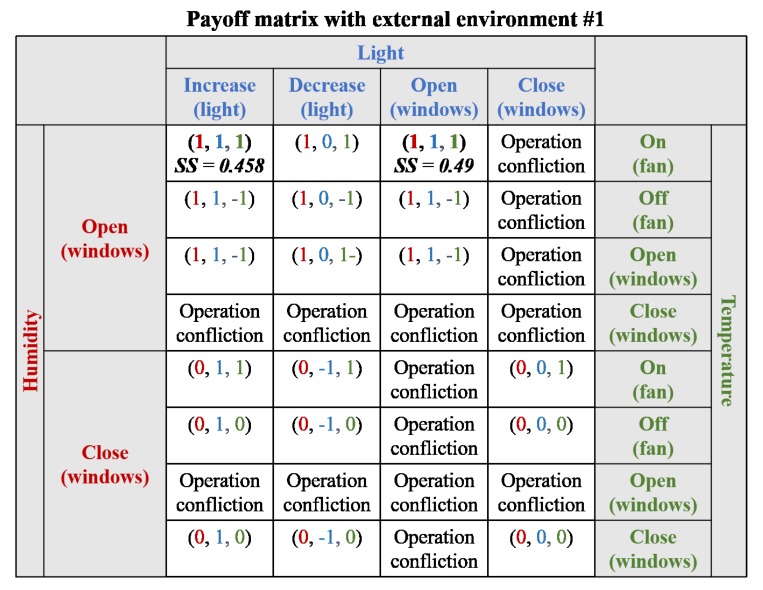
Payoff matrix for Scenario #3 with external environment #1 (α = 0.5 and β = 0.5).

**Figure 11:**
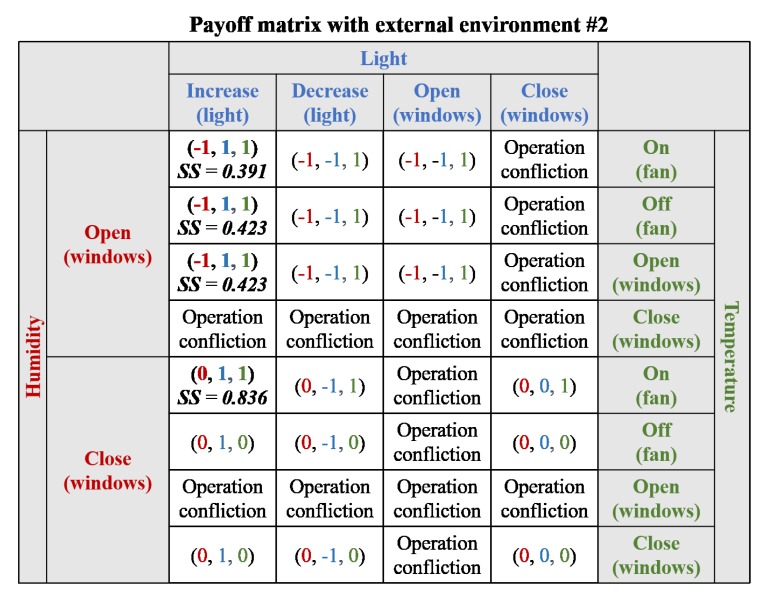
Payoff matrix for Scenario #3 with external environment #2 (α = 0.5 and β = 0.5).

In the third scenario, none of the requirements were satisfied. Therefore, the payoff matrix was associated with three requirements and related act-devices. A payoff matrix of the scenario is presented in Figure 10 and Figure 11. There were 14 conflicting operations related to the window and those operations were discarded in each payoff matrix. In the payoff matrix with the first external environment (See Figure 10), there were two strategies within the Nash equilibrium: “increase light controller, open windows and turn on fan” and “open windows and turn on the fan”. Both strategies could satisfy all requirements and the latter was selected as the most optimal strategy, considering the results of strategy score because the optimal strategy was satisfied by activating fewer act-devices. However, in the third scenario with the second external experiment, there was no Nash equilibrium for satisfying all requirements, and thus the candidate strategies with the largest number of Nash equilibrium were extracted, as shown in Figure 11. There were three candidate strategies for adaptation: “increase light, open windows and turn on fan”, “increase light, open windows, and keep fan turned off”, and “increase light, keep windows closed, and turn on fan”. All candidate strategies could satisfy the light and temperature requirements, but the humidity requirement was not satisfied; therefore, all candidate strategies included the same number of satisfied requirements (i.e., SR in the strategy score). However, the third candidate strategy included minimal act-device operations (i.e., AD in the strategy score) and lower related requirements (i.e., RR in the strategy score) by maintaining closed windows. Therefore, the third candidate strategy had the highest strategy score and was selected as the optimal solution.

The results of the case study present the proposed framework design models and the optimal solution chosen. As shown in the modeling results, the proposed model can determine the relationship between the devices and provide information (i.e., abstracted model) for runtime verification. In addition, the results of strategy selection illustrate the measurement of the effectiveness of the adaptive strategies.

## 6. Discussion

In this section, we present a discussion on the limitations of the proposed framework and the future work to overcome the limitations. First, we present a limitation and future work on modeling and decision-making. In the proposed framework, modeling classifies IoT devices as act-devices and read-devices, and those devices need related requirement information to design the system model. In addition, the relation between the devices and requirements is used in the decision-making method to determine the Nash equilibrium. Human intention or predefined information is required to determine the relationship between the devices and the requirements of the devices. In addition, there is no way to verify whether the designed relationships are really affected by each other. A relationship between the requirements via the operations of act-devices is crucial because, if the relationship is overlooked in the design phase, it can lead to wrong verification and decision-making. To address this limitation, we plan to propose an automation method to determine and verify the relationship between the IoT devices and the requirements to prevent overlooked system modeling.

The second limitation is related to the extraction of an adaptive strategy. In the proposed method, a game theoretic decision-making method is used to extract strategy candidates and the optimal solution. The decision-making method utilizes the status of act-devices and related environment data when adaptation is needed, and the adaptive strategies are generated to adapt to a specific moment. In other words, the results of decision-making are only considered as adaptations for situations in the present and not in the future. Therefore, the execution of the optimal solution can have a negative effect on the future. Accordingly, future adaptation must be considered in the decision-making method. To address this limitation, we plan to improve the decision-making method by estimating the changes that may occur in the future using machine learning. Particularly, the concept of reinforcement learning can be used to extract and make adaptive strategies.

The third limitation is related to the robustness of the decision-making method. In the proposed method, we assume that players (i.e., requirements) are rational players, which are legitimate users in Nash equilibrium. However, there may exist a malicious player who aims to increase the costs incurred by the rational players [51,52]. If malicious players exist in the proposed decision-making method, then an incorrect adaptation may occur by fabricating strategies (i.e., act-devices) and information for decision-making (i.e., sensor-devices). To address this limitation, we plan to improve the proposed decision-making method by considering malicious players in an IoT environment. In addition, to prevent the malicious player, we also consider the authentication and authorization methods in the design phase of the proposed framework.

The fourth limitation is related to the management of sensor-devices. In this paper, we focus on modeling, verifying, and decision-making for centralized IoT environment with low-computing devices. Hence, sensor-related optimization is pretermitted. However, generally an IoT environment consists of multiple sensor-devices and the sensing process is an important part of an IoT environment. Therefore, several optimization processes (e.g., merging, transmitting, etc.) may be considered in the proposed framework. To address the limitations of sensor optimization, we have an initial plan to improve the proposed finite-state machine model with sensor optimization. The verification and decision-making methods will be improved gradually.

## 7. Conclusions

Several IoT frameworks include various requirements to accomplish different objectives in a changing environment, and the requirements must be dynamically satisfied at runtime. To address this problem, we propose a self-adaptive framework for strategy extraction with verification in an IoT environment at runtime. The proposed framework comprises two phases: modeling and runtime. The modeling phase is responsible for finding an available IoT device and building the system model. To build the system model, a finite-state machine is proposed. After the modeling process, an abstracting process is executed to abstract the built model into an equation form using a state elimination algorithm [30,31]. The abstracted results are transferred to the runtime phase. The runtime phase includes a MAPE loop. In the monitoring phase, environmental data are collected from available sensor devices and transferred to the analyzing phase. The analyzing phase then calculates the equations extracted in the modeling phase and verifies the satisfaction of the requirements at runtime. In the planning process, the adaptive strategies are extracted using the proposed Nash equilibrium. The strategies are evaluated in the planning process and the most efficient strategy is executed in the execution process. In this study, the suitability of the proposed framework was demonstrated using experiments, which showed that the proposed framework can be applied at runtime, yielding reasonable results in terms of computation time. In addition, we propose a simple IoT-based smart green house to understand the procedures associated with modeling and strategy extraction of the proposed framework. Moreover, we present a discussion on the limitations of the proposed framework, including the future work to address the limitations discussed.

In future work, optimization of the proposed method will be performed, particularly the planning process, such that reinforcement learning can be applied to the proposed framework to implement it in a physical environment [53,54]. In addition, we will improve the proposed approach using an automation method to generate the relationships between the IoT devices and the requirements.

## Figures and Tables

**Table 1:** Comparison of previous research and the proposed model.

Worked by	Goal	Lifecycle	Approach
Zhang et al. [7]	Inventory management systems with self-adaptive distributed decision support models	Loop with update	- Artificial neural network for recognition of scenarios - Knowledge and rule-based decision-making
Lunardi et al. [8]	Automated decision analytics and support for IoT	Loop with update	- Rule-based reasoner
Mezghani et al. [10]	Autonomic cognitive design patterns for smart IoT-based systems	Loop with update	- Design patterns for cognitive IoT-based systems - Patterns that provide generic and reusable solutions
Ouechtati et al. [11]	A framework for access control in the IoT environment	Dynamic adaptation process (loop)	- Dynamic adaptive process based on risk value, politics, and rule sets
Muccini et al. [12]	A framework for IoT modeling	MAPE loop	- Analysis of IoT and self-adaptation control patterns - Bridge and combine the IoT distribution patterns and adaptation logic
Shekhar et al. [13]	A framework for adaptive resource management in cyber physical systems and IoT	Feedback loop	- Collect data from cloud and edge resources - Learn and enhance models - Apply enhanced models in a feedback loop
Azimi et al. [9]	A hierarchical computing architecture for IoT-based health monitoring system	MAPE-K loop	- A hierarchical computing architecture with cloud and fog enabled IoT - Closed loop for autonomic system - Machine learning data analytics
Ribeiro et al. [42]	Management of architectural patterns for self-adaptive system in IoT	MAPE loop	- An architectural pattern for self-adaptive systems with a control loop
Welsh et al. [43]	A self-adaptive system with goal-based requirements models to provide self-explanation at runtime	Loop with update	- Goal-based model - Simply structured domain-specific language
Beal et al. [44]	Aggregate programming with simplified design, creation, and maintenance for IoT software systems	N/A	- Aggregate programming abstraction layers - Field calculus constructs - Building-block APIs
Bucchiarone et al. [46]	A service composition framework with runtime service composition in a dynamic context	Loop with update	- Service model with stateful, non-deterministic, and asynchronous features
Renart et al. [14,48]	A data-driven framework to support dynamic data driven IoT applications	N/A	- Associative rendezvous interaction model- Simple rule-based abstraction with two different types of rules
Sylla et al. [47]	Self-adaptive framework design with multiple autonomic loops for reliability	MAPE-K	- Multiple autonomic loops
**Proposed**	**Self-adaptive framework for IoT**	**MAPE loop**	**- Finite-state machine modeling for IoT** **- Model-checking based runtime verification** **- Game theory based decision-making method**

**Table 2:** Details of hardware environments for performance measurement.

Hardware	CPU Clock (GHz)	Number of CPU Core	Memory (GB)	Operating System
Laptop (Intel i5-5200U)	2.7	2	8	Windows 10
Desktop (Intel i5-4670)	3.4	4	16	Windows 10
Server (Intel Xeon E3-1230L v3)	1.8	4	4	Windows 10
Samsung Galaxy S8	2.31	8	4	Android 8.0.0
